# A new toolset for protein expression and subcellular localization studies in citrus and its application to *citrus tristeza virus* proteins

**DOI:** 10.1186/s13007-017-0270-7

**Published:** 2018-01-09

**Authors:** Amit Levy, Choaa El-Mochtar, Chunxia Wang, Michael Goodin, Vladimir Orbovic

**Affiliations:** 1 Department of Plant Pathology, Citrus Research and Education Center, University of Florida, Gainesville, FL USA; 2 Citrus Research and Education Center, University of Florida, Lake Alfred, FL USA; 3 Department of Plant Pathology, University of Kentucky, Lexington, KY USA

**Keywords:** Citrus, Transient expression, Transformation, Cell biology, *Citrus tristeza virus*

## Abstract

**Background:**

Transient gene expression is a powerful tool to study gene function in plants. In citrus, *Agrobacterium* transformation is the method of choice for transient expression studies, but this method does not work efficiently with many gene constructs, and there is a need for a more robust transient expression system in citrus leaves. Biolistic particle delivery is an alternative to *Agrobacterium* transformation, and in some plants, such as Arabidopsis, gives higher transformation rates in leaf tissues than *Agrobacterium*.

**Results:**

Here we describe an improved method for gene expression in epidermal cells of citrus leaves, using the Bio-Rad Helios gene-gun. Gene-gun bombardment of GFP-HDEL produced highly efficient gene expression in large number of cells and in different citrus varieties. We show here that transiently expressed proteins have maintained their functions in plants, and this is demonstrated by the subcellular localization of different organelle markers, and by a functional assay of *Xanthomonas citri* effector AvrGF1. To further expand the available tools for subcellular localization studies in citrus, we also generated a new set of transgenic citrus plants that contain organelle markers labelling the nuclei, actin and endoplasmic reticulum. Using these new tools, we were able to show that the coat protein of *citrus tristeza virus* localizes to the cytoplasm and nuclei when expressed in epidermal cells fused to GFP.

**Conclusion:**

We have optimized a new method for transient expression in citrus leaves, to give highly reproducible and efficient transformation without producing a high level of injury or artifacts to the bombarded tissue. We also generated the first set organelle markers for use in citrus. These fluorescent protein markers label the nucleus and the actin. With these new resources, protein activity and subcellular localization can be studied in citrus rapidly and in high throughput. The handheld gene-gun device can also be used in the grove to deliver therapies for citrus diseases, such as canker and Huanglongbing, into trees.

**Electronic supplementary material:**

The online version of this article (10.1186/s13007-017-0270-7) contains supplementary material, which is available to authorized users.

## Background

Genetic engineering is becoming widely accepted as a solution for disease control in plants, including diseases in citrus. This is especially true for the expression of plant-derived genes, and for new methods of gene editing. Recent studies in genomics and plant pathology studies using citrus and other, especially model, plants have identified many more gene candidates that can be incorporated into other species to generate pathogen-resistant plants. For example, it was recently shown that the expression of NPR1 in citrus trees resulted in partial resistance/tolerance to diseases such as citrus canker and greening under field conditions [1]. When working with perennials such as citrus, generation of a genetically engineered plant is a long and laborious process [2, 3]. For this reason, there is a need to develop an efficient, reproducible and easy methodology for transient genetic transformation of citrus tissues in order to easily screen these candidates and identify genes that perform well and can be used to produce stably transformed plants. Moreover, tools to study citrus proteins properties, such as intracellular localization, are lacking. Transient gene expression can provide valuable data about various characteristics of these proteins, such as subcellular localization and intra/inter cellular trafficking, expression levels, stability and degradation, interactions with other proteins, and activity [for example, induction of hypersensitive response (HR)]. The development of an easy and efficient transient expression system in citrus will therefore undoubtedly speed up our investigations of gene function in this host plant [4].

In citrus, transient expression is carried out by two methods. In the first method, gene expression is delivered by *Agrobacterium* infection. *Agrobacterium* transformation was successfully applied to various citrus cultivars, and this method has been employed to study the type III effector gene *avrGf1* in grapefruit and pepper *Bs2* gene in *Citrus limon* [5, 6]. However, *Agrobacterium*-mediated transient expression has been especially difficult to use [7, 8]. Recently, a modification for this technique was described, in which *Xanthomonas citri* subsp*. citri* (Xcc) pre-treatment before agro infiltration dramatically enhanced transient β-glucuronidase (GUS) expression in leaves of six citrus varieties—Valencia sweet orange (*Citrus sinensis* var. Valencia), Duncan grapefruit (*Citrus paradisi*), Key lime (*C. aurantifolia* L.), Carrizo citrange (*C. sinensis* × *Poncirus trifoliata*), Sour orange (*C. aurantium*), and Meiwa kumquat (*Fortunella crassifolia*) [9]. *Agrobacterium* infection may carry a limitation by generating potentially unpredictable effects of bacterial effector proteins known to be exported into the plant cells together with the transforming T-DNA [10]. Adding Xcc pretreatment might even further complicate this undesirable effect, and influence the interpretation of the obtained results.

A second transient expression method is based on delivery of DNA by particle bombardment. Using this method with Carrizo citrange thin epicotyl segments, efficiencies of up to 93% of the bombarded plants, with an average of 102 GUS spots per tissue, have been reported [8]. Still, no efficient bombardment method has been described for citrus leaves, which is the preferable tissue for transient expression since it is represented by organs that are the largest and easiest to harvest, manipulate and observe [4].

Fluorescent proteins (FPs) tags are suitable for plant research especially for those integrated functional genomics projects where intracellular localization of proteins synthesized in response to infection by the pathogen is followed in respect to changes in the host gene expression. In this work, we describe a highly efficient method for the delivery of plasmid DNA into the epidermis of young citrus leaves, using the Bio-Rad Helios gene gun system [4]. This technique displayed a highly robust, efficient and reproducible transient expression of a variety of functional proteins with diverse biological activities and subcellular localizations. We also describe a new set of transgenic citrus plants that allow visualization of actin and the nuclei in citrus, and we demonstrate how these new lines can be combined with transient transformation for functional studies in citrus. Using these new tools, we were able to demonstrate that the coat protein (CP) of—*citrus tristeza virus* (CTV) co-localized with Histone-RFP in the nuclei of citrus cells. These new resources will provide an efficient new toolset for studying function and localization of proteins in citrus, and will enable more rapid citrus gene studies.

## Results

### Bio-Rad Helios gene gun system for robust transient expression in citrus

**Fig. 1:**
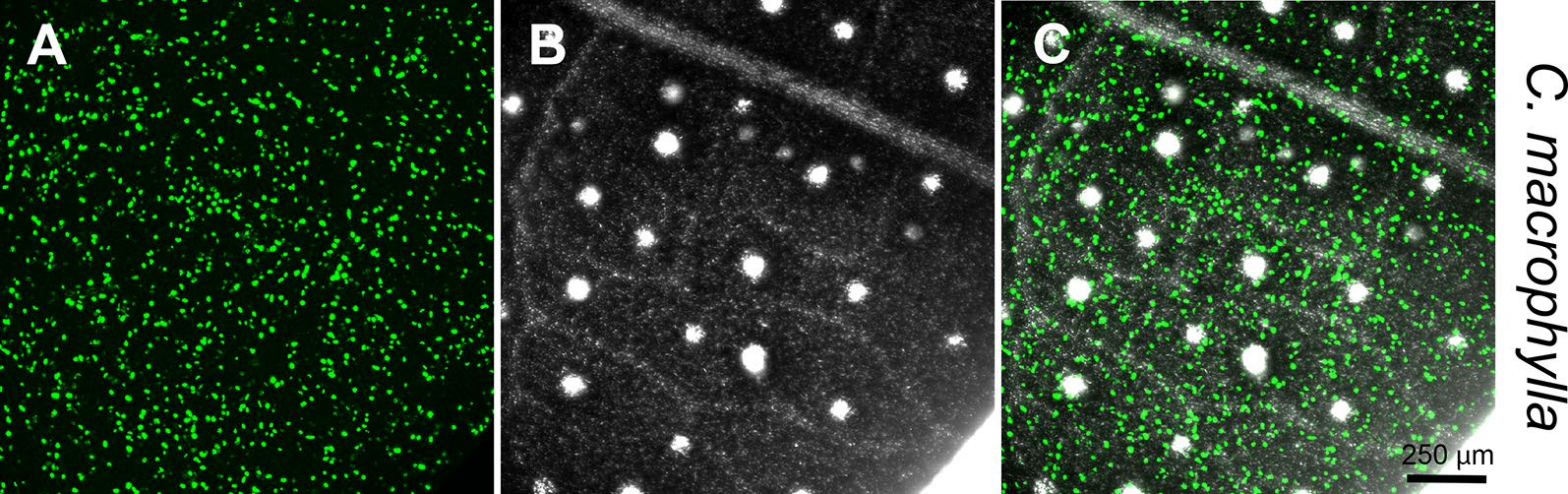
CLSM-projected Z-series of C-mac abaxial leaf section after bombardment with ER-GFP. Image was taken 40 h after bombardment. Green fluorescence (**a**), brightfield (**b**) and merged image (**c**) are shown. White areas in the brightfield image are oil cavities

We used the Helios gene-gun in order to develop an efficient transient-expression system for citrus leaves. In this technique, DNA-coated gold particles are precipitated on a plastic tube and then fired into the plant by helium pressure. Bombardment of an endoplasmic reticulum (ER) GFP marker (GFP-HDEL) into detached leaves of *Citrus macrophylla* (C-mac) resulted in up to ~ 700 GFP expressing cells in a typical 10 × magnification microscope field (Fig. 1). This highly efficient level of expression was obtained when young (2nd–3rd from the branch tip) leaves were used. In older leaves with better-developed cuticle, expression rate decreased dramatically. Bombardment was carried out most efficiently with high concentrations of both DNA and 0.6 µm gold particles (5 µg/shot and 0.5 mg/shot, respectively). Lowering the concentration of both DNA and gold particles decreased the average number of expressing cells in both C-mac; however, these differences were not statistically significant (Additional file 1). Surprisingly, lower gold concentration resulted in a statistically significant reduction of expression rate in Madam Vinous sweet orange (*Citrus sinensis* (L.) Osbeck) (Additional file 1), highlighting the level of variability found with particle bombardment between different varieties, and different bombardment events, which is a limitation of the bombardment method.

**Fig. 2:**
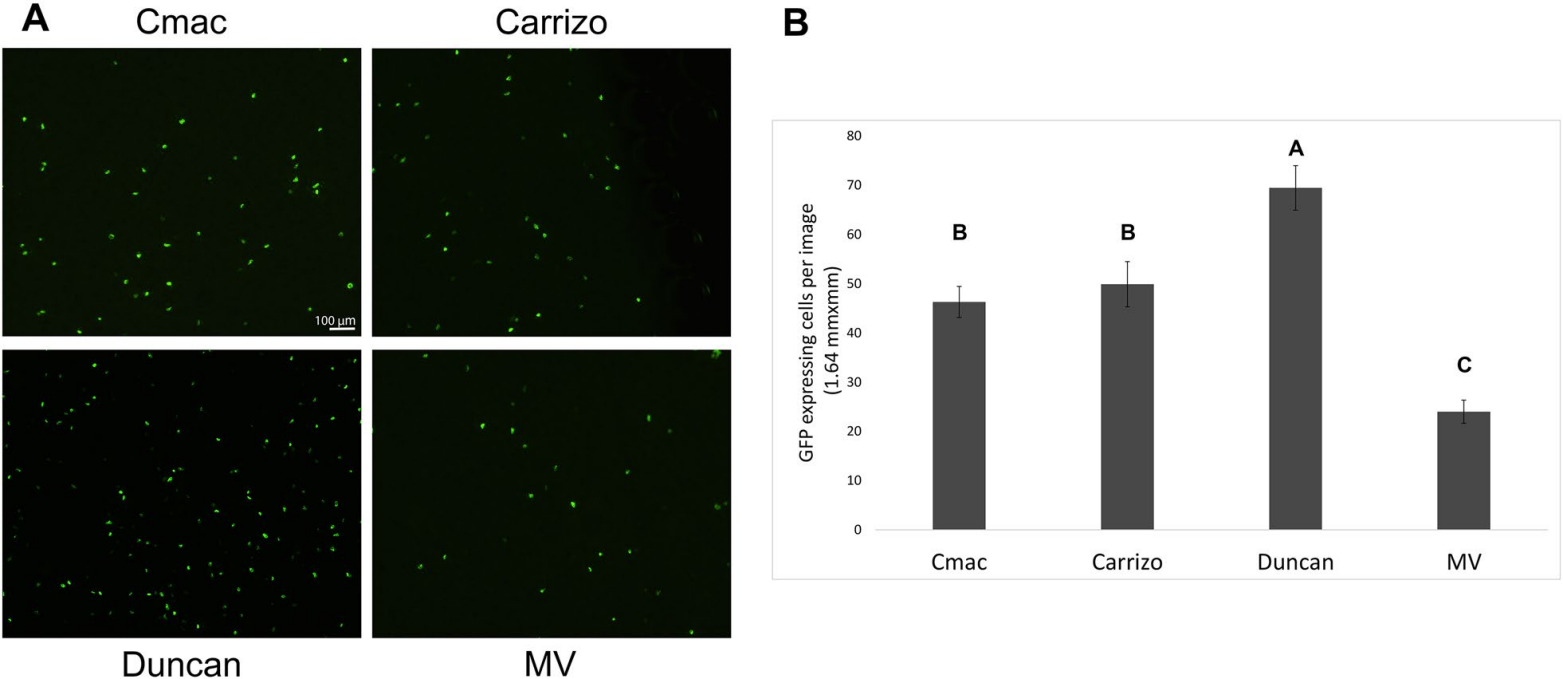
Bombardment efficiency in four different citrus varieties. **a** Fluorescence leaf cells after bombardment with ER-GFP, **b** average number of fluorescent cells in an image (1.64 mm^2^). Results are average of the average expression level in 10 bombardment events (10 leaves). Letters represent the statistically significant groups as determined by Tukey HSD test. Cmac—*Citrus macrophylla*, Carrizo—Carrizo citrange (*C. sinensis* × *Poncirus trifoliata*), Duncan—Duncan grapefruit (*Citrus paradisi*) and MV—Madam Vinous sweet orange (*Citrus sinensis* (L.) Osbeck). SE is shown in bars

We compared the expression levels of GFP-HDEL in four citrus varieties—C-mac, Carrizo citrange, Duncan grapefruit and Madam Vinous sweet orange. Forty hours after bombardment, GFP expressing cells were present in leaves from all four varieties, indicating that this method of transformation is not restricted to a specific citrus variety (Fig. 2). Bombardment efficiency, however, was different between varieties, with the number of GFP expressing cells being highest in leaves of Duncan grapefruit, and lowest in leaves of Madam Vinous sweet orange. The number of fluorescent cells in a 10 × image in C-mac and Carrizo citrange, was identical. In Madam Vinous, expression levels were about half of C-mac and Carrizo levels, and in Duncan grapefruit about 50% higher (Fig. 2).

### Localization and activity of transiently expressed proteins in citrus

**Fig. 3:**
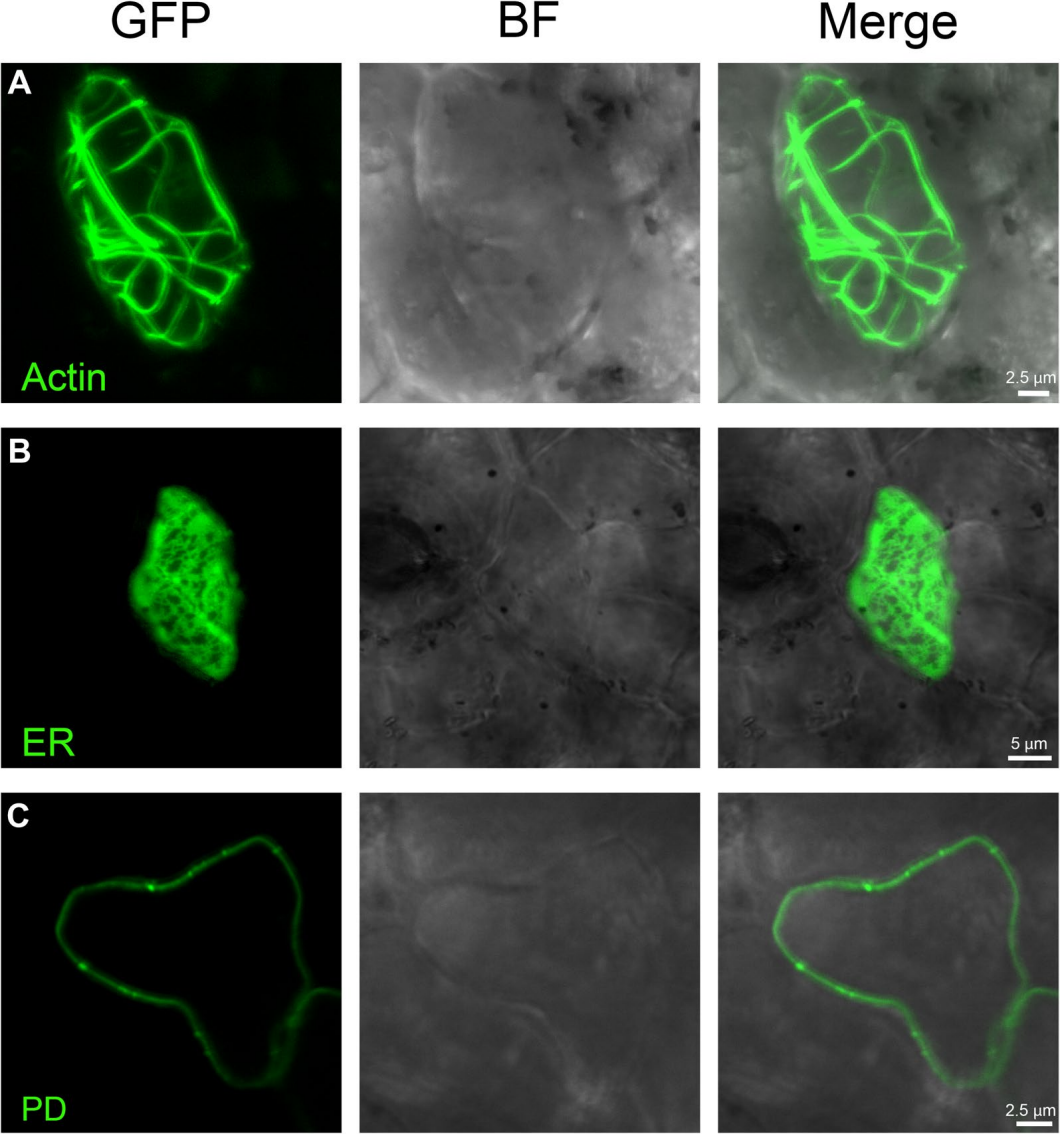
CLSM images of C-mac abaxial epidermal leaf cells after bombardment with UtrCH-GFP (**a**), GFP-HDEL (**b**) and AtBG_pap:GFP (**c**). Images show green fluorescence (left panel), brightfield image (centre) and merged image (right panel)

We determined the effectiveness of transient expression method in order to conduct functional studies of proteins with diverse biological activities and subcellular localization specificities. First, we expressed three different proteins that localize in different cellular organelles: (1) ER protein GFP-HDEL, (2) actin protein UtrCH-GFP and (3) plasmodesmata protein AtBG_ppap. GFP-HDEL is retained in the ER lumen [11]. UtrCH-GFP binds to actin through the calponin binding domain of utrophin (UtrCH) a probe reported to mark F-actin without altering the balance of actin assembly/disassembly [11]. AtBG_ppap is a beta-1,3-glucanase that targets the plasmodesmata after attachment to a glycosylphosphatidylinositol (GPI) plasma membrane anchor [12]. Forty hours after bombardment into C-mac leaves, these three markers were clearly localized at their expected target organelles (Fig. 3). Actin filaments labelled with UtrCH:GFP appeared highly bundled (Fig. 3). These results indicate that basic function of studied proteins (such actin binding, GPI anchoring etc.) are well preserved when transiently expressed in citrus by bombardment (Fig. 3).

**Fig. 4:**
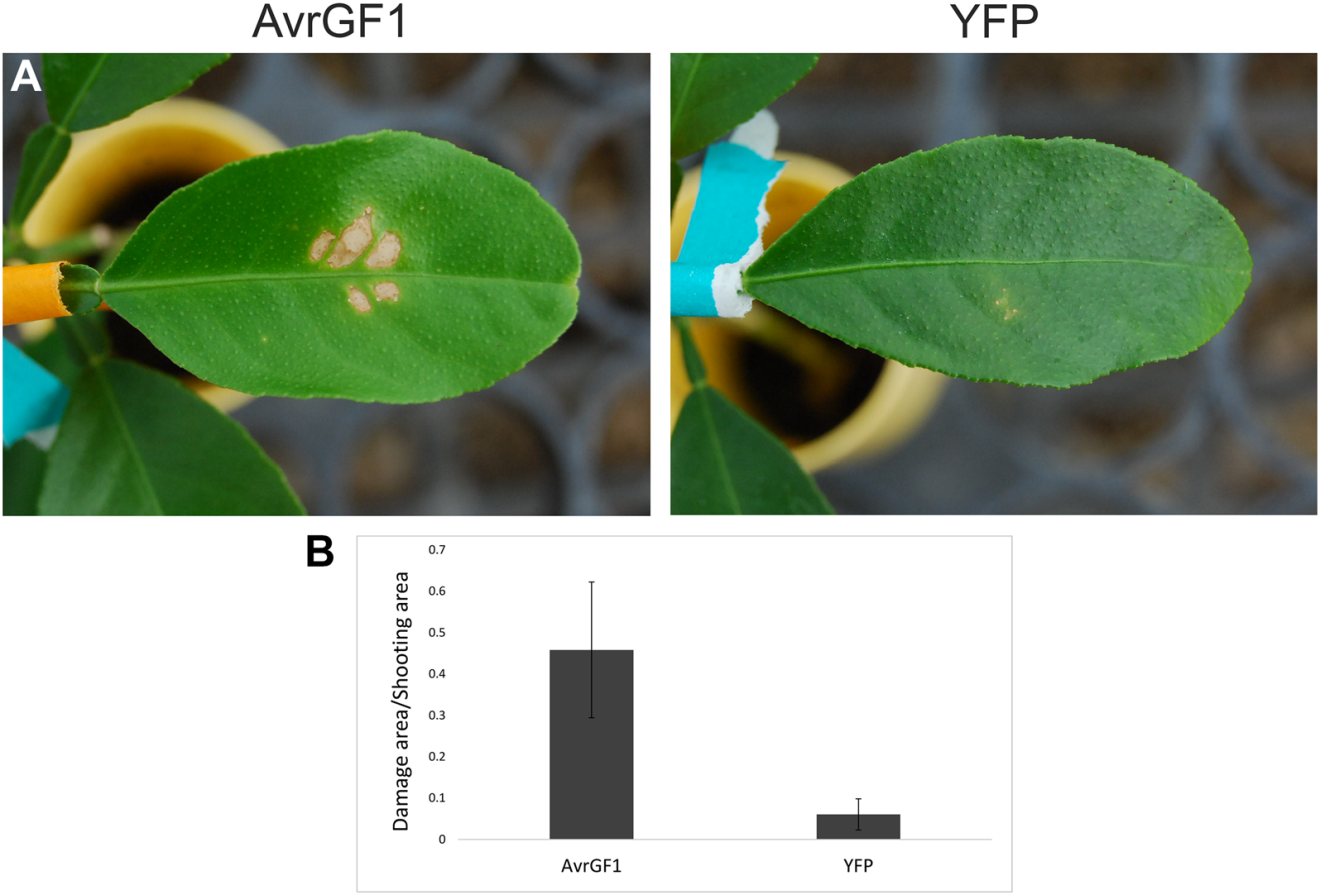
Expression and activity of *X. citri* AvrGf1 in C-mac leaves by particle bombardment. Young (2nd–3rd) intact leaves were bombarded with either AvrGF1 or YFP, both under the 35S promoter. **a** Representative images of the leaf cell death caused by either AvrGF1 or YFP, 7 days after bombardment, **b** average ratio between area of tissue damage and the gene-gun shooting area (4.91 cm^2^) on the leaves. Results are average of 12 bombardment events (12 leaves) (T-test; *P* < 0.05). SE is shown in bars

To further determine the biological functionality of genes introduced via bombardment, we used *AvrGF1* from *Xanthomonas citri*. AvrGF1 protein is a bacterial effector that was shown to induce an hypersensitive response (HR) when expressed in leaves of grapefruit (*Citrus paradisi*) by *Agrobacterium* inoculation [5]. In this assay, we bombarded young leaves of intact plants. Seven days after bombardment into leaves of C-mac, AvrGF1 expression resulted in the appearance of clear HR cell-death symptoms in the inoculated areas. Bombardment of yellow fluorescent protein (YFP) gene, on the other hand, resulted in minimal bombardment-associated damage, but did not result in HR phenotype (Fig. 4). Altogether, our results show that protein activity is maintained with this transient expression assay.

### Generation of transgenic citrus with labelled organelles

**Fig. 5:**
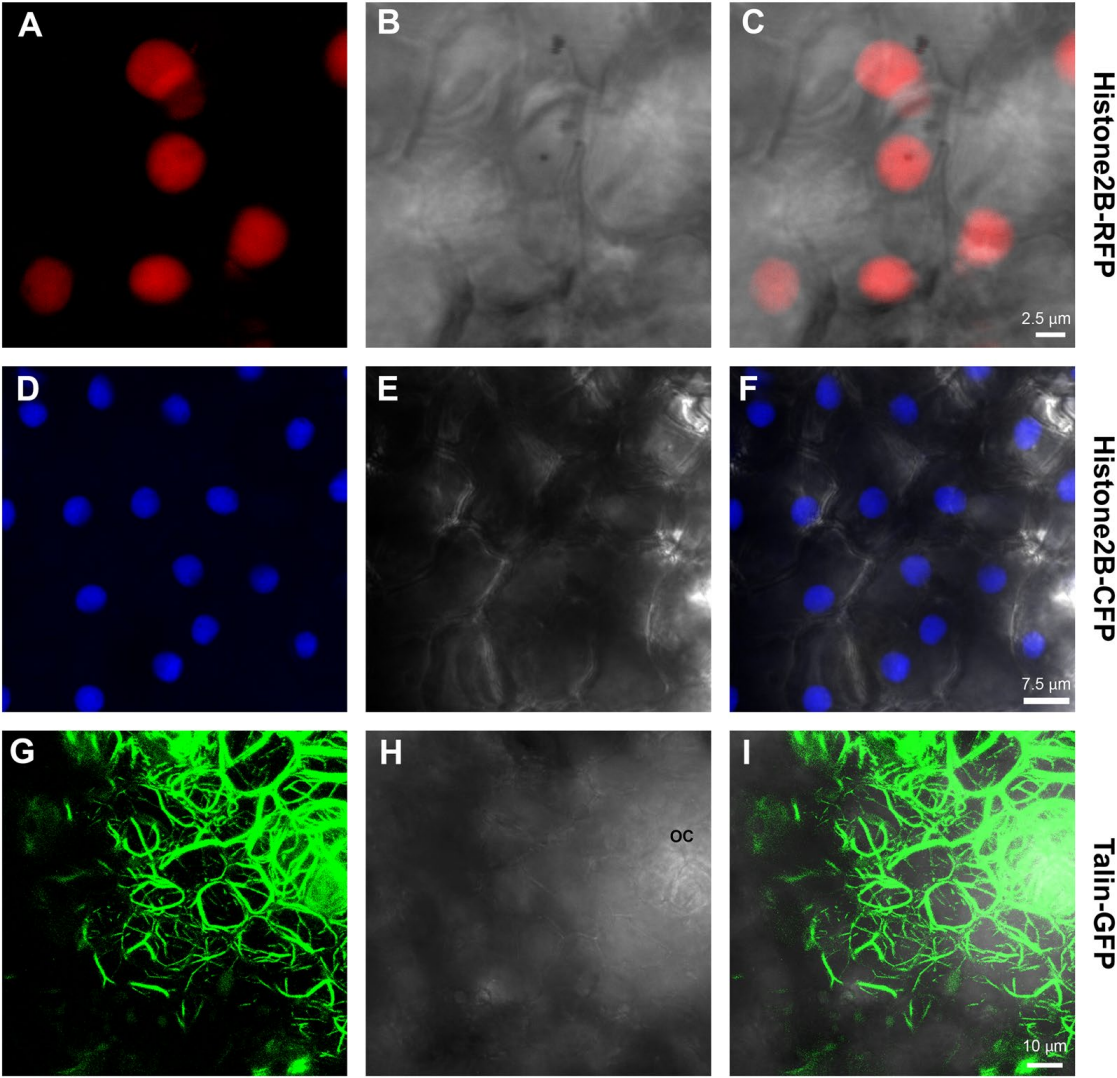
CLSM-projected Z series of abaxial transgenic Duncan grapefruit leaf epidermal cells stably expressing either Histone2B-RFP (**a**–**c**), Histone2B-CFP (**d**–**f**) and Talin-GFP (**g**–**i**). Fluoresence (**a**, **d**, **g**), brightfield images (**b**, **e**, h) and merged images (**c**, **f**, **i**), are shown. *OC* oil cavity

In order to further demonstrate the functionality of the bombardment method and to develop the resources available for functional gene assays in citrus, we generated transgenic plants of Duncan grapefruit and Carrizo citrange, in which ER, actin and nuclei were labeled with three different fluorescent proteins fusions. Stable incorporation of any of the four genes into the genomes of citrus plants did not induce any phenotypic changes, as transgenic plants looked similar to wild-type plants for the duration of this study when some of them were already 3 years old. Actin was labeled with Talin:GFP, ER was labelled with RFP-KDEL and two different AFP variants (CFP and RFP) fused to Histone2B were used to label nuclei [13]. Nuclear markers clearly displayed fluorescence of the fusion protein in the expected subcellular compartment (Fig. 5). Similar to the transiently expressed actin marker UtrCH:GFP (Fig. 3), Talin:GFP stably labelled actin which also appeared in a bundled form, and was extremely condensed in the oil cavities (Fig. 5). RFP-ER stable lines clearly labelled the nuclear envelope and high amounts at the cell periphery. However, the polygonal network of the ER did not display clearly, probably because of RFP low expression levels of the ER (and lower quantum yield of RFP compared to GFP). As a result, we were unable to expose the intricate structure of ER within the cells, and could not use these transgenic plants as accurate ER marker lines (data not shown).

### The coat protein of CTV localizes to nucleus

**Fig. 6:**
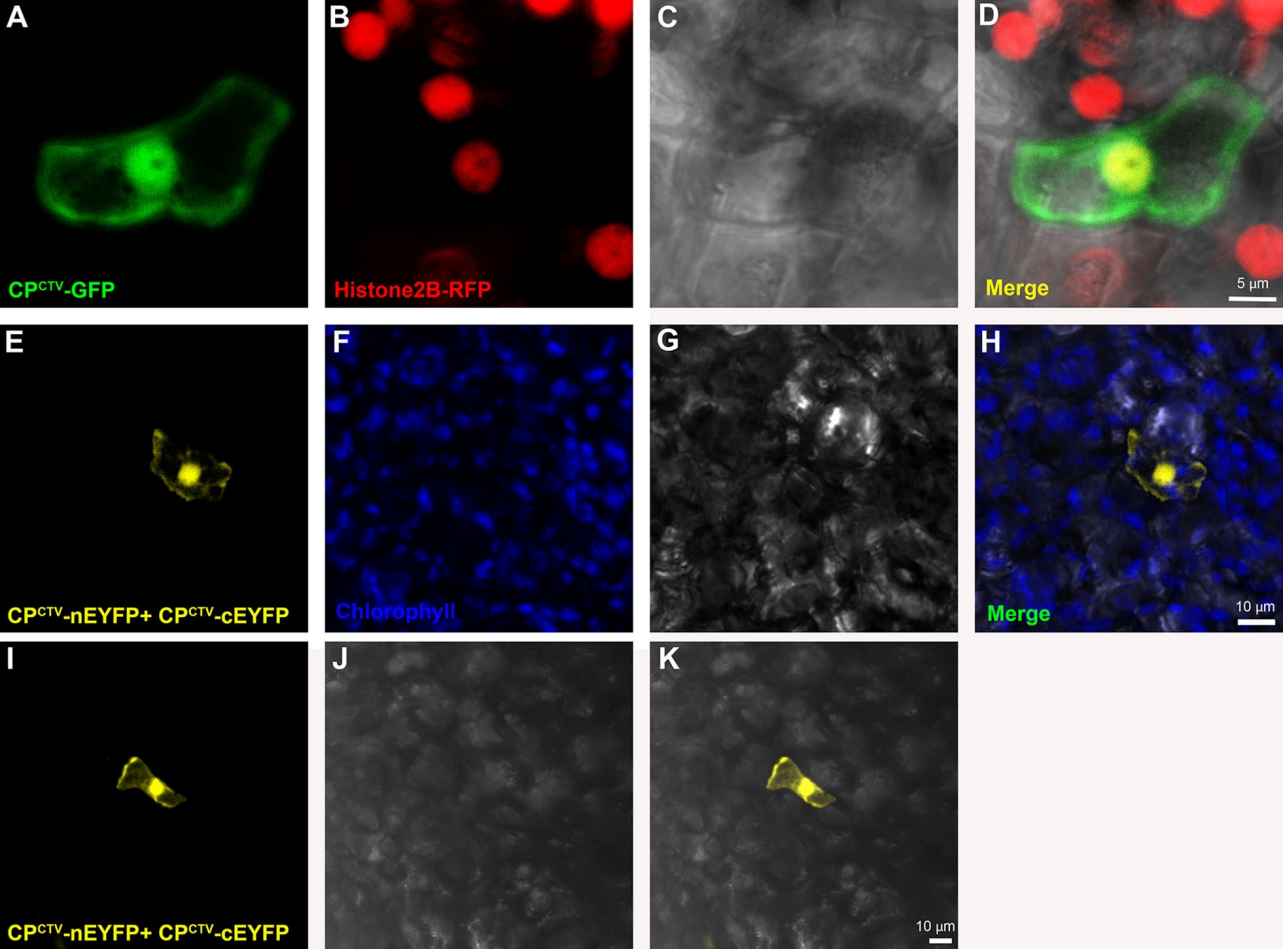
CP^CTV^ localizes to the cytoplasm and nucleus. **a**–**d** CLSM image of CP^CTV^–GFP after bombardment onto epidermal cells of transgenic Duncan grapefruit expressing Histone2B-RFP. CP^CTV^–GFP (green, **a**), Histone2B (red, **b**), brightfield image (**c**) and the corresponding superimposed image (**d**), are shown, **e**–**k** CLSM image of two cells showing BiFC YFP fluorescence after bombardment of CP^CTV^-nEYFP and CP^CTV^-cEYFP into epidermal cells of C-mac leaves. YFP fluorescence (**e**, **i** yellow), chlorophyll channel (**f**, blue), brightfield image (**g**, **j**) and superimposed image (**h**, **k**), are shown

We used these new resources in order to study the subcellular localization of the coat protein (CP) of CTV fused to GFP (CP^CTV^–GFP) in citrus. First, we bombarded CP^CTV^–GFP fusion under the control of a 35S promoter into citrus using the gene-gun. Forty hours after CP^CTV^–GFP gene was bombarded into C-mac, we could detect the protein in the cytoplasm of epidermal cells, and in what looked like the nuclei of the cells (Additional file 2). This expression pattern was similar to that of unfused YFP (Additional file 2). Next, we used the transgenic Histone 2B lines in order to verify the nuclear localization. We bombarded CP^CTV^–GFP gene into transgenic Duncan grapefruit plants that express Histone2B:RFP fusion. In these plants, CP^CTV^–GFP co-localized with Histone2B-RFP, indicating that CP^CTV^–GFP was indeed in the nucleus (Fig. 6). To further verify that nuclear localization fluorescence is not the result of GFP cleavage, we used the bimolecular fluorescence complementation (BiFC) method, in which CP^CTV^ is not fused to full-length YFP. Instead, fusions of CP^CTV^ with nEYFP and cEYFP [14] were co expressed from a single plasmid. Bombardment of this construct into C-mac resulted in the same expression cytoplasmic and nuclear pattern that was seen for CP^CTV^–GFP (Fig. 6). These results suggest that beyond its structural role in the formation of CTV capsids, CP^CTV^ may have another activity that involves an uncharacterized nuclear stage.

## Discussion

Here we describe a method for the efficient delivery of plasmid DNA into the epidermis of citrus leaves by microparticle bombardment, using the Bio-Rad Helios gene gun system. The main advantages of this system are: (1) it gives a high transformation efficiency (high number of transformed cells); (2) it is highly reproducible; (3) it does not produce significant damage or artifacts; (4) it is broadly applicable to different types of functional assays; and (5) it is broadly applicable to different citrus varieties. In addition, the gene gun has an important advantage over the traditional bombardment methods in the fact that it does not need any vacuum applied. The gun can be carried and used with any target material in the plate, greenhouse or field, with or without detaching the leaves, and this can be employed in order to deliver therapies directly into leaves of citrus plants in the grove. The technique described here, having all these characteristics, will therefore be suitable for functional studies of various citrus proteins with diverse biological activities and subcellular localizations, and for gene delivery in the field.

The main limitation of this method is that it is relatively expensive. Both the gene-gun and the gold particles are expensive, compared to the inexpensive *Agrobacterium* inoculation procedure. In addition, the bombardment does cause some damage to the bombarded tissue (see Fig. 4), although by avoiding the damaged area, thousands of undamaged transformed cells can be observed/used for intended purposes. A third limitation is that this method works much more efficiently with very young leaves, at least under the conditions described here. When older, but still not fully developed leaves were used, efficiency decreased dramatically, probably because the conditions were not suitable for sufficient particle penetration into the leaf. The last limitation is that there is variability between the experiments, which is characteristic of all bombardment methods. Despite all above-mentioned limitations, this method still generates reliable and robust transient expression in citrus, resulting in extremely high level of transformation, and therefore should be the method of choice when possible.

A major importance of transient expression systems is that it enables us to study protein function in transgenic or mutant citrus backgrounds. For example—studying protein localization in a transgenic plant where known organelle markers are expressed fused to FPs. To demonstrate this possibility, we generated three new citrus transgenic lines that express markers for nuclei and actin filaments. A forth marker for the ER did not display the expected results. Using these lines in combination with the gene-gun delivery method, we were able to carry out co-localization studies, and show that CP^CTV^:GFP co-localizes with Histone2B:RFP in the nuclei of citrus cells. The combination of the bombardment and our new transgenic lines that express fluorescent protein markers thus can offer an important support in experiments aimed at defining protein localization. Moreover, our stable transgenic lines can enable us to carry long-term experiments to explore cellular dynamics, and these experiments are currently conducted for CTV. To our knowledge, these types of studies were not available before for perennial plants. Recent works have demonstrated that different plant viral proteins target the nucleus. The role of the nucleus, and specifically the role of transcriptional reprogramming in the systemic movement of plant viruses, is an emerging theme [15]. For example, the movement protein of Tobamovirus *tomato mosaic virus* (ToMV) was shown to interact with transcription factors KELP and MFB1 [16, 17], and the one from *Turnip vein clearing virus* (TVCV) associated with nuclear F-actin-containing filaments [11] that were hypothesized to be involved in transcription regulation [18]. The crucifer strain of tobacco mosaic virus (TMV-cg) has been shown to modulate the expression of a WRKY8 transcription factor [19]. Recently it was also shown that TMV enhances its access to the phloem of mature plant tissues through the targeted disruption of auxin/indole acetic acid (Aux/IAA) transcriptional regulators that control expression of host genes involved in virus cell-to-cell movement, plasmodesmata gating, and defense [20]. The possible biological importance of CP^CTV^ in the nuclei is still unknown, but may involve interaction with host transcription factor. CP^CTV^ does not contain any nuclear localization signals in its sequence, and its entry into the nuclei may be by diffusion as a result of its small size. We used BiFC in order to demonstrate that nuclear localization is not the result of a cleavage in the fluorescent protein. However, we cannot completely rule out the rare scenario in which both nEYFP and cEYFP are cleaved from the fusions, and bind each other. Nevertheless, our BiFC expression strongly suggest CP^CTV^ self-interacts inside the nuclei, and thus points to a functional activity of CP^CTV^ in the nuclei. Future experiments will need to address this questions.

Finally, the possibilities to conduct functional studies to test candidate genes in citrus are very limited, while generating transgenic plants is an extremely long and laborious process [3]. One of the major promises of the method described here is that it will enable researchers to meet the challenges facing the citrus industry from citrus canker to citrus greening by conducting rapid functional studies in citrus to test protein expression and activity, before generating transgenic plants. In addition, the handheld gun can be used in order to deliver therapies directly to the plant leaves in the grove.

## Conclusions

We have optimized a new method for transient expression in citrus leaves, which is based on the Bio-Rad Helios gene-gun bombardment. This method is highly reproducible, gives a high transformation efficiency, and does not produce a high level of injury or artifacts to the bombarded tissue. This method routinely resulted in transformation of hundreds of cells in a typical microscope field at 10 × magnification. We have used subcellular localization studies, and HR induction to show that proteins that are expressed using this method maintain their activities in planta. To further expand the toolset for functional assays in citrus, we also generated a new set of transgenic citrus organelle marker lines, with markers to the nucleus, actin and ER. Bombardment of the CP of CTV into the nucleus marker line determined that CP^CTV^ localizes in the cytoplasm and nucleus when expressed fused to GFP. Diseases such as citrus canker and citrus greening are threatening citrus production worldwide. The idea of using genetic engineering in order to provide the needed solutions is becoming more acceptable, but progress is slowed down by the inability to conduct quick functional studies in citrus to test candidate genes when generating transgenic plants is an extremely long and laborious process. The new tools and resources described here can help conduct functional assays with diverse proteins and citrus varieties, and can speed up the race for greening and canker solutions.

## Methods

### Plant material and plasmid constructs

All plants used in this study were grown in the Citrus Research and Education Center (lake Alfred, FL), and maintained under greenhouse conditions. Construction of GFP-HDEL, RFP-HDEL, UtrCH-GFP, AtBG_pap-GFP, Talin-GFP, Histone-RFP and Histone-CFP have been described previously [12, 13, 21]. To express AvRGF1 under the 35S promoter, the entire coding sequence of AvrGF1 was PCR amplified directly from *X. citri* cells grown in liquid culture overnight, using the primers 5′-GGGGACAAGTTTGTACAAAAAAGCAGGCTTCATGGCTCCGAGCATGCATTC-3′ and 5′-GGGGACCACTTTGTACAAGAAAGTGGGTCTTAGTCGCTGCTGGTCATTG-3′. PCR product was purified, cloned into pDONR207, and then into pSITE0A destination vector [22].

For generating CP^CTV^–GFP, we used overlap extension PCR [23] to build a hybrid gene consisting of a 5′UTR of tobacco etch virus as a translational enhancer fused to the 5′ end of the CTV T36 strain coat protein ORF followed by a linker sequence and the 5′ end of EYFP ORF, using primers C-1728: 5′-AACACAACATATACAAAACAAACGAAT-3′, C-1863: 5′-CACCATTTACGAACGATAGCAATGGACGACGAAACAAAGAAATT-3′, C-1864: 5′-AATTTCTTTGTTTCGTCGTCCATTGCTATCGTTCGTAAATGGTG-3′, C-1856: 5′-TAT CCATGGTTAGTACAGCTCGTCCATGCCGAGAGTGAT-3′, C-1858: 5′-AGATCAATAGCCACG ATGGTGAGCAAGGGCGAGGAGCTGTT-3′ and C-1866: 5′-TCACCATCGTGGCTATTGATCTACGTGTGTTGAATTTCCCAA-3′. Cloning was done into pCASS4N (a variant of pCASS2 that contains NcoI [24]) between the StuI and NcoI restriction sites.

The CTV CP–CP interacting BiFC (Bimolecular fluorescence complementation) plasmids were constructed in three steps using standard molecular biology protocols. The first step included cloning the CP ORF into pSAT1A-nEYFP-N1 using primers 5′-TATCTCGAGATGGACGACGAAACAAAGAA-3′ and 5′-TATCCCGGGACGTGTGTTGAATTTCCCAAGCT-3′, and pSAT4-cEYFP-C1(B) using primers 5′-GATCTCGAGCCATGGACGACGAAACAAAGAA-3′ and 5′-TATCCCGGGAAACGTGTGTTGAATTTCCCAAGCT-3′ [14]. The CP PCR as well as both plasmids were digested with SmaI and XhoI, respectively. The second step was to mobilize these plasmids into pPZP-RCS1 [25]. The gene cassette of CP in plasmid pSAT4-cEYFP-C1(B) was mobilized into MBA-38 after digesting both plasmids with I-SceI. pPZP-RCS1 was alkaline phosphatase treated to prevent self-ligation. The third step was to use the plasmid generated in step 2 to introduce the gene cassette of CP in pSAT1A-nEYFP-N1 to pPZP-RCS1 carrying the expression cassette of pSAT4-cEYFP-C1(B). Both plasmids were digested with AscI, and the appropriate fragments were ligated.

### Particle bombardment into citrus leaves

For bombardment, all plasmids were first purified from *E. coli* with the Qiagen plasmid maxi-kit (Qiagen, Hilden, Germany). Particle bombardment was carried out as described in [4], with few modifications. Gold particles coating with DNA was performed as described in [4], but only 0.6 µm particles were used, mixed with 150 µg of DNA for the preparation, to give ~ 5 µg of DNA per bullet. Gold concentration was 0.5 mg/shot, unless noted otherwise. Bombardment was carried out on the abaxial side of the leaf, using the 2nd and 3rd newest leaves of the citrus plants. Finally, delivery into the plants was carried out with a helium pressure of 260–280 psi. After bombardment, leaves were kept in the dark inside a petri dish with wet filter paper, to maintain humid conditions. In all the experiments described here, we used a single cartridge per leaf. However, the size of citrus leaves is usually big enough for multiple shots if needed for functional studies.

### Transformation

Transgenic plants that belong to Carrizo citrange rootstock cultivar and Duncan grapefruit cultivar were produced according to well established protocol that employed *Agrobacterium tumefaciens* [3]. Starting material in transformation experiments were juvenile explants cut from stems of germinated seedlings. All transgenic shoots were micro-grafted onto Carrizo rootstock and allowed to grow in vitro for 4–6 weeks. The plantlets consisting of growing transgenic shoots and Carrizo roots were moved from tubes to 2′ × 2′ × 2′ pots kept under plastic dome on the light bench in the laboratory for additional 2 months. Subsequently, plants were moved to greenhouse and re-planted into bigger pots.

Selection of transgenic shoots carrying gene for GFP-Talin fusion was done by visualizing GFP. Transgenic plants expressing genes for three other fusion proteins were selected in the PCR-based screen [3]. The sequences of PCR primers used for selection of transgenic shoots are presented in Table 1. Pieces of tissue from the shoots that were selected as putative transformants in PCR screen were observed under confocal laser-scanning microscope to confirm synthesis/proper attachment of fusion proteins.

**Table 1:** The sequences of PCR primers used for selection of transgenic shoots

Primer	Sequence
CFP-Histone 2B (f)	AGCTGACCCTGAAGTTCATCTG
CFP-Histone 2B (r)	GATATAGACGTTGTGGCTGATGTAG
RFP-Histone 2B (f)	CGTAATGCAGAAGAAGACCA
RFP-Histone 2B (r)	CTTGATGTCGGTCTTGTAGG
RFP-ER (f)	CCGACTACTTGAAGCTGTCCTTC
RFP-ER (r)	GTACTGTTCCACGATGGTGTAGT

### Microscopy

Selection of transgenic shoots carrying gene for GFP-Talin fusion was done using a Leica Wild M3Z stereomicroscope (Leica Microsystems Inc., Buffalo Grove, IL, USA) outfitted with a NIGHTSEA™ Stereo Microscope Fluorescent Adaptor (NIGHTSEA, Lexington, MA, USA) and the filter set for Royal Blue (excitation 400–415 nm with a 460 nm longpass emission filter). GFP expressing cells on different citrus varieties were imaged using an Olympus BX61 epi-fluorescence microscope and an OMAX A35140U 14MP CMOS camera. Fluorescence was captured using a FITCI dichroic cube. All other CLSM images were collected using a Leica SP8 laser-scanning confocal microscope (Leica Microsystems Inc., Buffalo Grove, IL, USA). Samples were imaged using lasers with excitation wavelengths for their respective fluorescent reporting range: GFP fluorescence was excited with a 488-nm argon laser, and emission was detected at 500–530 nm. RFP fluorescence was excited with a 561-nm diode-pumped solid-state (DPSS) laser, and emission was detected at 590–630 nm. Cyan fluorescent protein (CFP) was excited with near UV diode 405-nm laser, and emission was detected at 475–501 nm. Leica Application Suite—LAS X (Leica Microsystems Inc., Buffalo Grove, IL, USA) was used to collect z-stacks composed of optical sections at a 1024 × 1024 resolution. Images were exported as TIFF files to produce photographs for publication using Adobe Photoshop software.

### Statistical analysis

Statistical analysis of images was preformed using the JMP Pro 13.2 Statistical program (SAS, Cary, NC). Differences in gold concentration, plasmid concentration and AvrGF1 HR response were analyzed using T-test, while the difference between varieties was analyzed using the Tukey HSD.

## Additional files

**Additional file 1.** Relation between expression efficiency and DNA/Gold particle concentration. Graphs of the average number of fluorescent cells in a 10 × image area after bombardments with different gold particle concentrations and plasmid DNA concentrations.

**Additional file 2.** CLSM images of CP^CTV^–GFP and free YFP after bombardment into epidermal cells of C-mac. (A-C) CLSM image of CP^CTV^–GFP (A, green), brightfield image (B) and superimposed image (C). (D-F) CLSM image of unfused YFP (A, yellow), with brightfield image (B), Chlorophyll channel (C, Blue) and superimposed image (D).
