# Supplementary material for: A new toolset for protein expression and subcellular localization studies in citrus and its application to citrus tristeza virus proteins
Source: Plant Methods. 2018 Jan 9;14:2. doi: 10.1186/s13007-017-0270-7 (PMC5759842; doi:10.1186/s13007-017-0270-7)
Supplement: Supplementary file 1 — Additional file 1. Relation between expression efficiency and DNA/Gold particle concentration. Graphs of the average number of fluorescent cells in a 10 × image area after bombardments with different gold particle concentrations and plasmid DNA concentrations. [file 13007_2017_270_MOESM1_ESM.docx]

**Additional file 1: Comparison of gold and plasmid concentrations**

1. Average number of fluorescent cells in a 10x field in *Citrus macrophylla* (Cmac) and Madam Vinous sweet orange (MV) after Bombardment of YFP with two with different gold concentrations. Lower gold concentration reduced average number of fluorescent cells, however results are statistically significant for MV (*P* < 0.001) but not for Cmac (*P*=0.138). SE is shown in bars.
2. Average number of fluorescent cells in a 10x field in *Citrus macrophylla* (Cmac) after Bombardment of YFP with two with two plasmid DNA concentrations. Lower DNA concentration reduced average number of fluorescent cells, however results were not statistically significant for MV (*P* =0.88). SE is shown in bars.
