# Supplementary figures and images for: A new toolset for protein expression and subcellular localization studies in citrus and its application to citrus tristeza virus proteins
Source: Plant Methods. 2018 Jan 9;14:2. doi: 10.1186/s13007-017-0270-7 (PMC5759842; doi:10.1186/s13007-017-0270-7)

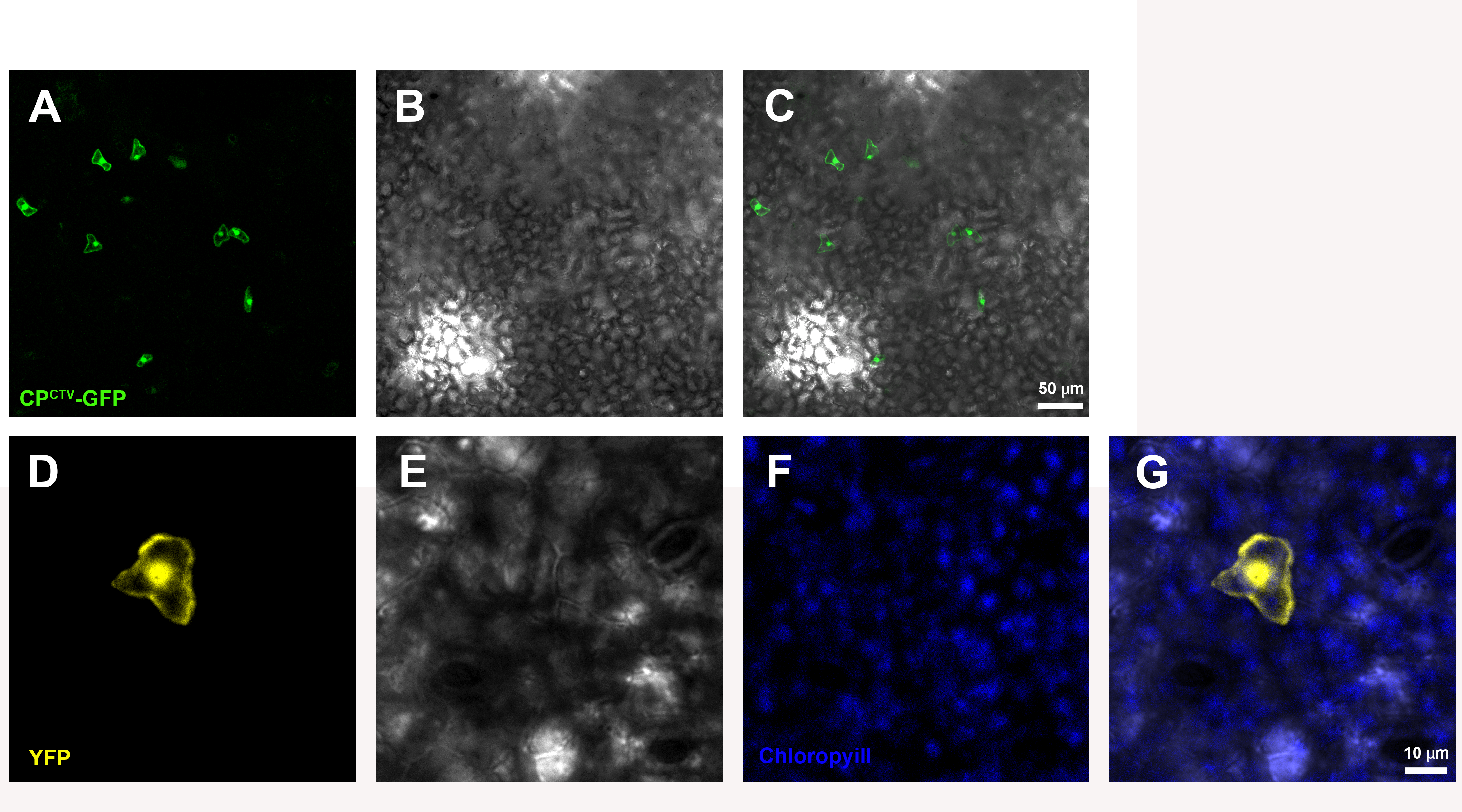

Supplement: Supplementary file 2 — Additional file 2. CLSM images of CPCTV–GFP and free YFP after bombardment into epidermal cells of C-mac. (A-C) CLSM image of CPCTV–GFP (A, green), brightfield image (B) and superimposed image (C). (D-F) CLSM image of unfused YFP (A, yellow), with brightfield image (B), Chlorophyll channel (C, Blue) and superimposed image (D). [file 13007_2017_270_MOESM2_ESM.tif]
